# Supplementary material for: Nanopore sequencing reveals endogenous NMD-targeted isoforms in human cells
Source: Genome Biol. 2021 Aug 13;22:223. doi: 10.1186/s13059-021-02439-3 (PMC8361881; doi:10.1186/s13059-021-02439-3)
Supplement: Supplementary file 1 — Additional file 1. Figures S1 and S2 and the uncropped Western blots in Figure S3. [file 13059_2021_2439_MOESM1_ESM.pdf]

Supplementary Figures

Nanopore sequencing reveals endogenous NMD-targeted isoforms in human cells

Evangelos D. Karousis, Foivos Gypas, Mihaela Zavolan and Oliver Mühlemann

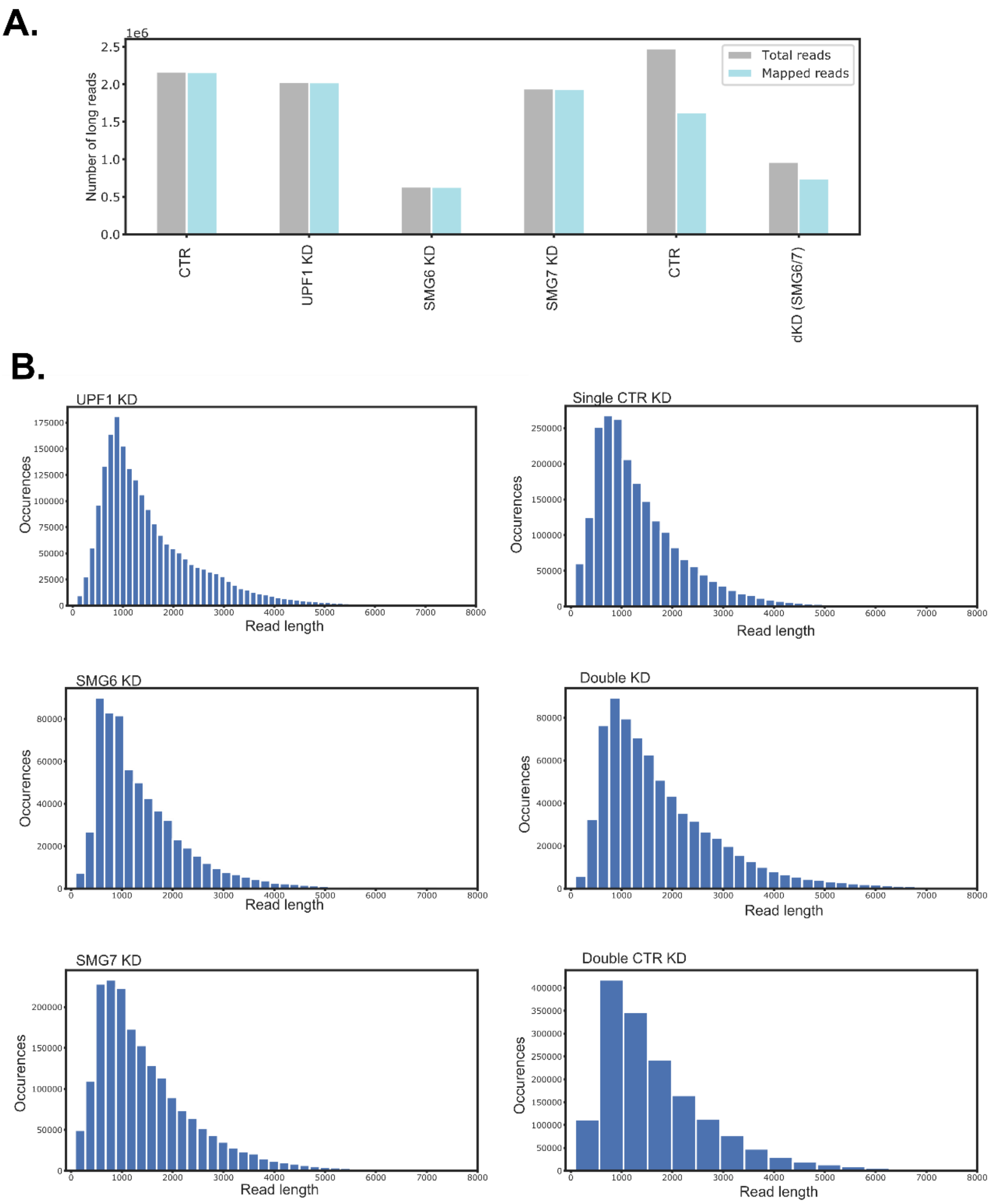

Figure S1 (A) Number of totally sequenced (grey) and mapped Nanopore reads (light blue) in the different cDNA sequencing experiments of reverse-transcribed total RNA from cells with the indicated knockdowns. **(B)** Read length distribution of mapped reads from the long-read cDNA sequencing experiments indicated in (A).

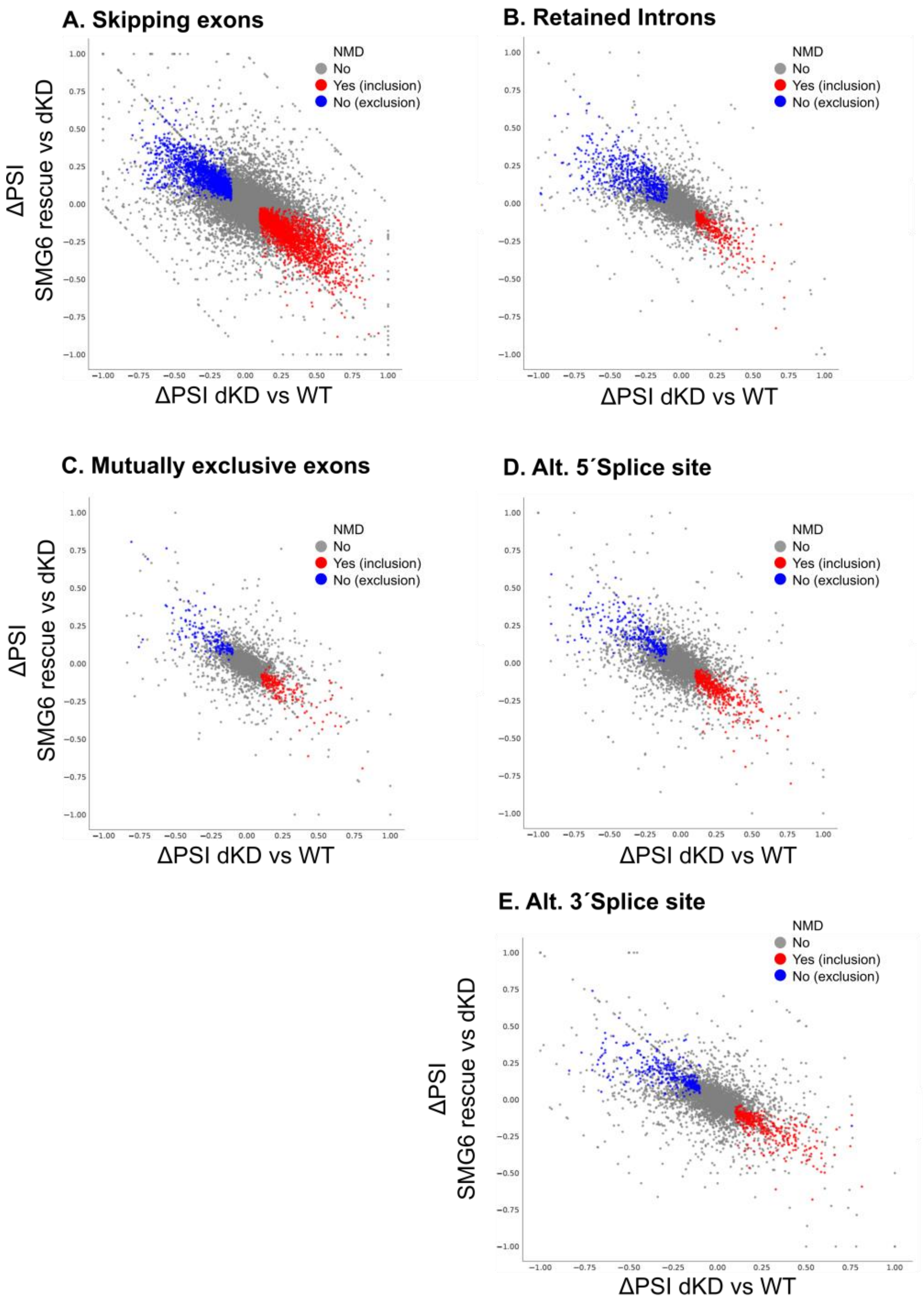

Figure S2 (A-E) Scatter plots comparing the change in "percent spliced in" (deltaPSI) of exons in CTR versus dKD (*x*-axis) and SMG6 rescue versus dKD (*y*-axis). Coloured in red are significant exon inclusion and in blue significant exon exclusion events. Non-significant events are depicted in grey.

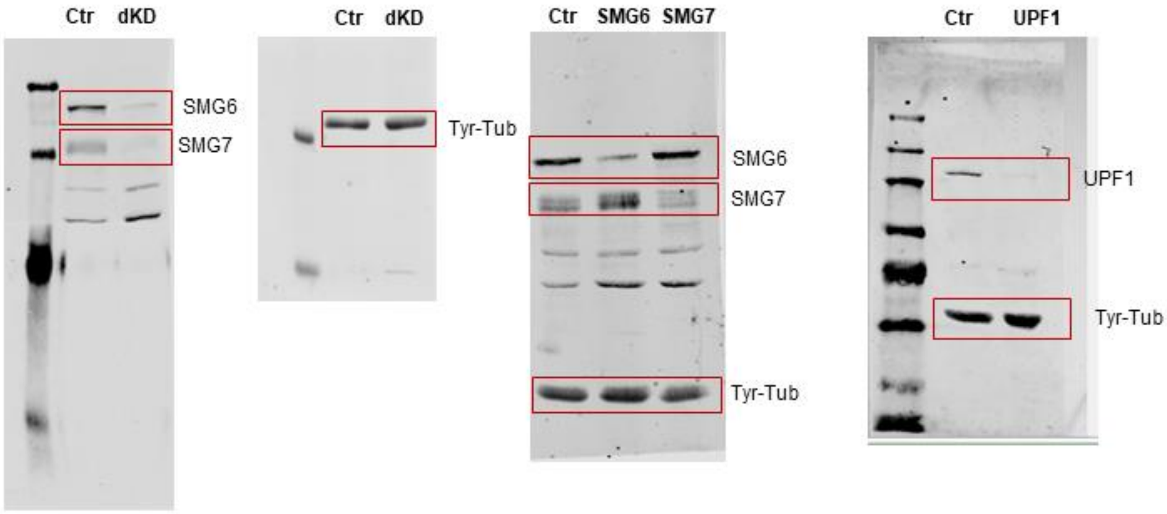

Figure S3 Uncropped Western blots
