# Supplementary material for: Nanopore sequencing reveals endogenous NMD-targeted isoforms in human cells
Source: Genome Biol. 2021 Aug 13;22:223. doi: 10.1186/s13059-021-02439-3 (PMC8361881; doi:10.1186/s13059-021-02439-3)
Supplement: Supplementary file 3 — Additional file 3. Review history. [file 13059_2021_2439_MOESM3_ESM.docx]

Review History

**First round of review**

**Reviewer 1**

**Are you able to assess all statistics in the manuscript, including the appropriateness of statistical tests used?**

No, I do not feel adequately qualified to assess the statistics.

**Comments to author:**

In this interesting study Karousis and colleagues introduce a new experimental approach for the identification of RNA targets that are sensitive to degradation by the Nonsense-mediated decay (NMD) pathway. This has been a long-standing issue in the field and this manuscript provides important new tools to address this problem. The authors used a combination of cDNA Nanopore sequencing together with short-read sequencing in human cell line that have altered levels of NMD factors. The authors depleted UFP1, SMG6 or SMG7 individually or in combinations from HeLa cells using shRNAs, following an experimental design used by them in the past (Colombo et al. (2018).

To circumvent the major limitation of long-read sequencing related to its moderate sequencing depth, the authors chose to combine Nanopore long-read sequencing combination with Illumina short-read sequencing. An obvious advantage of this experimental approach is the ability to identify full-length NMD targets that become evident only upon inhibition of NMD. In particular this new approach was shown to be very efficient at the identification of NMD substrates that originate as a consequence of Alternative splicing (AS-NMD).

Using this new experimental set up, the authors were able to revisit features that are present in NMD-sensitive mRNAs. They confirmed that many NMD targets derived from exon skipping events and also observed the importance of exon junctions in the 3'UTR. By contrast, they found that the length of 3'UTR is not correlated with NMD sensitivity (Fig 4C), which was surprising.

Overall, this is a very interesting study that provides a new tool for the identification of NMD substrates. I would predict that this strategy will be widely used in the field. I do recommend publication in Genome Biology.

Specific comments

- An interesting, yet surprising finding of this manuscript is the lack of influence of the 3'UTR length in determining NMD sensitivity. This feature was inferred in previous papers by Muhleman and Lykke-Andersen labs, among others, mostly using NMD reporters. What is not clear from the Discussion is whether the 3'UTR length was also seen as an NMD-inducing feature in genome-wide studies using short reads. This should be discussed.

- Long reads originated in this study were combined with short reads from a similar experiment previously published by the same authors, where they carried out an almost identical experiment. It would have perhaps been better to use both long reads and short reads from the same samples originated in this study.

- It is not clear what data is presented in some figures. For example, does Figure 2E describe combined data form all KD experiments, or common NMD targets?

- It would be informative to describe the long-read seq data in more detail. How do UPF1 targets compare to SMG6/7? One of the main findings of this study is that NMD removes mRNAs arising by erroneous processing. Do these newly identified isoforms tend to be similar, or are there distinct isoforms predominantly regulated by individual NMD factors?

- It would be interesting that the authors discuss the role of NMD in transcriptional versus post-transcriptional gene expression regulation. Is it possible that targets previously identified as transcriptionally regulated due to 'intronic' reads are in fact previously un-annotated isoforms and reflect post-transcriptional gene expression regulation?

Minor

- When referring to the expression of neoantigens in disease, the authors may wish to refer to one of the first papers describing this phenomenon (Pastor et al (2010) Nature | PMID: 20463739)

- I don't think that Ref 23 relates to inhibition of NMD. Please double check. This is a paper from the Kornblihtt lab dealing with AS and UV irradiation

- Exon skipping should be referred to as alternative exons.

**Reviewer 2**

**Are you able to assess all statistics in the manuscript, including the appropriateness of statistical tests used?**

Yes, and I have assessed the statistics in my report.

**Comments to author:**

Karousis et al. use long-read sequencing in NMD-deficient cells to identify new NMD-targeted isoforms, and use this novel data to better examine the relative importance of NMD-triggering features in transcripts in human cells. The study is overall interesting, timely and well-executed. I have some suggestions for minor revisions:

1- P13 L43 - "This result indicates that uORFs and exon-exon junctions downstream of the stop codon are orthogonal features conferring NMD-sensitivity the enrichment of uORFs in NMD-sensitive mRNAs only becoming apparent in mRNAs lacking exons in the 3 UTR." Consider rephrasing for clarity. Related to this, did they check if this uORFs enrichment was absent in NMD-sensitive with exons in 3'UTR?

2- P7. "NMD targets specific RNA isoforms". With the overall approach to identify NMD targets they are probably including some indirect NMD targets as well. Those might be the NMD exon skipping events where the expression changes only in the gene (Fig 2E, black dots) but not in the transcripts. If that is the case, then it may not be surprising to see exon skipping as the main NMD-inducing type of event, because it's also the most common splicing event in normal genes. Can they comment on this possibility that NMD indirect targets confound analysis to inflate the relative frequency of exon-skipping as the NMD inducing event?

3- P13 L13 - "we decided to address the longstanding question of whether NMD also targets endogenous mRNAs with long 3 UTRs, independently of exon junctions." [...]

Their data, and other previous analysis (e.g. PMID: 27618451) suggest that 3'UTR length seems not to be an NMD-inducing feature in human. However there are also prior studies with experimental work showing that the longer the 3' UTR, the higher degradation by NMD, even without 3' UTR EJC (although this is not as efficient as the isoforms having 3' UTR EJC), possibly due to the quantity of UPF1 protein that is bound to the 3' UTR. Can they comment on what would be the reason for having observed the effects of 3' UTR lengths previously? Perhaps the UTR-length triggering only happens in certain transcripts, but it is not a general rule. Is it possible that in Fig 4C, we only observe a significant difference in the first <2000 nt or so?

4- Supp Fig 1A. what happened with SMG6 an dKD conditions? They obtained 75% less reads than in the other samples. Do they have replicates? I understood they have 3 replicates for the Illumina sequencing, but no replicates for the Nanopore. In these two conditions they detect a similar number of genes/transcripts as in the other samples/conditions (Fig.1C), so it might mean that the coverage is 75% lower if I understand correctly. Is this decreasing the reliability of the SMG6&_dKD results?

5- P19 L31: "it should be taken into consideration that many computationally predicted uORFs are in fact not translated and therefore do not stimulate NMD (52)." Could they consult a database with evidence of translation of various transcripts in human, such that they could filter out all the uORFS for which translation is not reported. This would help gain confidence that the higher differences in uORFs abundance in NMD-sens vs NMD-insens transcripts is due to translation of uORFs.

6- could they comment on and/or quantitate whether the long-read sequencing technology is overall more important for the discovery of new NMD target isoforms (with very low gene expression and so missed thus far), or for the refinement of already known NMD targets?

Other remarks:

- regarding P8 L29 and P7 L51, clarification may be needed. From how I understood the text, the newly detected isoforms are in 686 of 8056 genes, which accounts for ~8% of the genes (or exon skipping events?) with no significant gene expression change. If that is not the case, which is the total number (and % total genes/transcripts) of newly identified isoforms? The text also mentions a 13%, but this seems to refer to the newly-identified splicing events.

- P22 L21 - "the tool selects one start codon for each gene from the APPRIS (103) database of high-confidence transcript isoforms and then looks for a downstream in-frame stop codon in the transcripts annotated for the given gene" I presume all of the stops are normal stop codons (not PTC); if otherwise, consider rephrasing.

- References 85 and 93 appear duplicated.

- Page 4, line 25-26. Consider rephrasing sentence.

- Fig. 1B. legend could be improved, e.g. they mention the blue and green lines, but not the purple lines. In the lower part (representation of the short-reads expression level patterns of NMD-sensitive exons) - I found it a bit confusing why if they are NMD-sensitive exons, in the right plot they are downregulated under NMD KD conditions. I suppose that the each plot represents a different isoform of the same transcript: the isoform on the left contains a NMD-sensitive exon and the isoform on the right does not contain this exon. They might want to explain.

- They could explain better what the NMD KD rescue condition entails. They show it in figures and refer to it through the text, but give no explanation how they do the rescue in the methods. We just know from the text that they overexpress the NMD factors; more experimental detail would be appreciated.

- Fig. 2E - in the legend they should state which two conditions they are comparing (I think it's dKD vs control).

- Fig. 4D - they claim that NMD-sensitive transcripts have more uORFS than NMD-insensitive transcripts (in the text and in the discussion). According to Fig. 4d, it is 35% vs 45%. I think they should mention these percentages in the text. From reading the text, one gets the impression of striking differences, but in reality the differences are subtle. (described by the word "evident", which is, admittedly, technically not wrong for a 10% difference).

- Fig. 5 - besides the individual dots, it may be helpful to depict the distribution of the SRSF group (pink) to highlight the different behaviour from the rest of NMD splicing sites.

- Discussion is a bit verbose, and reads more like a summary of the paper. I would consider dropping some parts e.g. pg.18 line 27: they explain again the 50 nts rule (redundant).

**Authors Response**

**Point-by-point responses to the reviewers’ comments:**

*We would like to thank the editor for the invitation to revise our manuscript according to the reviewer comments and the reviewers for their careful and positive evaluation of our work and for the constructive feedback, which we have used as the basis for revising our manuscript. Below please find our point-to-point response to all issues raised by the two reviewers.*

Reviewer #1:

In this interesting study Karousis and colleagues introduce a new experimental approach for the identification of RNA targets that are sensitive to degradation by the Nonsense-mediated decay (NMD) pathway. This has been a long-standing issue in the field and this manuscript provides important new tools to address this problem. The authors used a combination of cDNA Nanopore sequencing together with short-read sequencing in human cell line that have altered levels of NMD factors. The authors depleted UFP1, SMG6 or SMG7 individually or in combinations from HeLa cells using shRNAs, following an experimental design used by them in the past (Colombo et al. (2018).

To circumvent the major limitation of long-read sequencing related to its moderate sequencing depth, the authors chose to combine Nanopore long-read sequencing combination with Illumina short-read sequencing. An obvious advantage of this experimental approach is the ability to identify full-length NMD targets that become evident only upon inhibition of NMD. In particular, this new approach was shown to be very efficient at the identification of NMD substrates that originate as a consequence of Alternative splicing (AS-NMD).

Using this new experimental set up, the authors were able to revisit features that are present in NMD-sensitive mRNAs. They confirmed that many NMD targets derived from exon skipping events and also observed the importance of exon junctions in the 3'UTR. By contrast, they found that the length of 3'UTR is not correlated with NMD sensitivity (Fig 4C), which was surprising.

Overall, this is a very interesting study that provides a new tool for the identification of NMD substrates. I would predict that this strategy will be widely used in the field. I do recommend publication in Genome Biology.

*Response: Thank you for the positive evaluation of our work.*

Specific comments

- An interesting, yet surprising finding of this manuscript is the lack of influence of the 3'UTR length in determining NMD sensitivity. This feature was inferred in previous papers by Muhleman and Lykke-Andersen labs, among others, mostly using NMD reporters. What is not clear from the Discussion is whether the 3'UTR length was also seen as an NMD-inducing feature in genome-wide studies using short reads. This should be discussed.

*Response: In the Colombo et al. paper, which was entirely based on short-read sequencing and thus had limited accuracy for conclusions at transcript level, we found that the fraction of NMD-sensitive transcripts that had annotated neither 5’ uORFs nor exon-exon junctions in 3’ UTRs had on average slightly longer predicted 3’ UTRs, but with a limited statistical power. In the present manuscript, in Fig. 4C, we find that when we exclude transcripts with exon-exon junctions in the 3’ UTRs the difference in 3’ UTR between NMD-sensitive and insensitive isoform is very small and not significant. Although both analyses gave rather similar result, its conclusions are different, which we think is an important argument for using as accurately as possible annotated isoforms. We discussed this in the manuscript and have added the following sentence to emphasize the previously different conclusion (p. 19):*

*“Notably, in our previous study that was based on short-read sequencing reads we observed that NMD-targeted genes have overall a longer 3΄UTR but with a limited statistical significance (Colombo et al., 2017).”*

- Long reads originated in this study were combined with short reads from a similar experiment previously published by the same authors, where they carried out an almost identical experiment. It would have perhaps been better to use both long reads and short reads from the same samples originated in this study.

*Response: The reviewer correctly points out that we used here the short read data form our previous study by Colombo et al. When Nanopore sequencing finally became available, we decided to complement, expand and improve our previous study with long-read sequencing data and repeated the experiments under the exact same experimental conditions and with the same cell lines. Given the high reproducibility and quality of the short-read data, we did not think it would have been cost-effective to redo the short-read sequencing.*

- It is not clear what data is presented in some figures. For example, does Figure 2E describe combined data form all KD experiments, or common NMD targets?

*Response: Indeed, this analysis (as well as other gene/transcript expression results) was done by combining the data of all replicates from dKD of SMG6 and SMG7 and rescue with SMG6. To clarify this point, the legend for Fig. 2E was revised as follows:*

*“Scatter plot depicting the log2 fold change (log2FC) of NMD-sensitive transcripts (y-axis) versus the log2FC between dKD and CTR of their corresponding genes (x-axis) showing alternative exon events (Ν= 8'056) based on SMG6/SMG7 double KD, CTR KD and SMG6 Rescue conditions.”*

- It would be informative to describe the long-read seq data in more detail. How do UPF1 targets compare to SMG6/7? One of the main findings of this study is that NMD removes mRNAs arising by erroneous processing. Do these newly identified isoforms tend to be similar, or are there distinct isoforms predominantly regulated by individual NMD factors?

*Response: We primarily looked at the overlap of SMG6/7 targets, because we expected the factors to act on similar targets, which is indeed what we observed (Fig. 2D), whereas UPF1 in addition to its role in NMD is also involved in other mRNA decay pathways. However, if we overlay the UPF1 targets, we also see a strong overlap with those. However, UPF1 targets are fewer in numbers because the achieved rescue condition was less efficient than for SMG6, resulting in fewer transcripts reaching the set threshold as shown in the Venn diagram below:*

*For this reason, we focused on the SMG6 targets for the downstream analyses but we included the long reads from the UPF1 condition to the transcriptome of reference. Also as expected, almost half of the 733 exons that responded to UPF1 KD (338; 46%) were not affected by SMG6/SMG7, consistent with the previously reported involvement of UPF1 in NMD-unrelated RNA decay pathways.*

*During the revision of our manuscript, we also realized that for Fig. 2D, only exons that were upregulated under dKD conditions were taken into consideration, while the exons that are reduced under dKD conditions and enriched upon rescue with SMG6 or SMG7 were omitted, which is incorrect. We now updated Fig. 2D with the correct numbers that include both the up- and the downregulated exons. Except for the increased numbers, nothing changed and all conclusions drawn from this figure remain unchanged.*

- It would be interesting that the authors discuss the role of NMD in transcriptional versus post-transcriptional gene expression regulation. Is it possible that targets previously identified as transcriptionally regulated due to 'intronic' reads are in fact previously un-annotated isoforms and reflect post-transcriptional gene expression regulation?

*Response: This is possible, although this has not been directly addressed in the literature, where intronic reads are assumed to come from all transcripts, not only a subset that would be NMD targets. However, to address it, we would have to analyze studies in which the level of intronic reads change, to find out whether the corresponding transcripts are NMD targets and whether the NMD factors change their expression levels. This would be a large and laborious analysis that goes beyond the scope of our manuscript.*

Minor

- When referring to the expression of neoantigens in disease, the authors may wish to refer to one of the first papers describing this phenomenon (Pastor et al (2010) Nature | PMID: 20463739)

*Response: We thank the reviewer for pointing out this study, and we have cited it in the revised manuscript (p.9, citation 23).*

- I don't think that Ref 23 relates to inhibition of NMD. Please double check. This is a paper from the Kornblihtt lab dealing with AS and UV irradiation

*Response: Thanks for carefully checking our citations and spotting this mistake, which we corrected by removing this citation.*

- Exon skipping should be referred to as alternative exons.

*Response: We agree with the reviewer that “alternative exons” is the more accurate term and we have therefore changed the terminology to alternative exons throughout the manuscript and in the figures.*

Reviewer #2: Karousis et al. use long-read sequencing in NMD-deficient cells to identify new NMD-targeted isoforms, and use this novel data to better examine the relative importance of NMD-triggering features in transcripts in human cells. The study is overall interesting, timely and well-executed. I have some suggestions for minor revisions:

1- P13 L43 - "This result indicates that uORFs and exon-exon junctions downstream of the stop codon are orthogonal features conferring NMD-sensitivity the enrichment of uORFs in NMD-sensitive mRNAs only becoming apparent in mRNAs lacking exons in the 3 UTR." Consider rephrasing for clarity. Related to this, did they check if this uORFs enrichment was absent in NMD-sensitive with exons in 3'UTR?

*Response: We have split this sentence into two sentences to improve clarity. It now reads: “This result indicates that uORFs and exon-exon junctions downstream of the stop codon are orthogonal features conferring NMD-sensitivity. That is, uORFs account for NMD-sensitivity of mRNAs that lack exon-exon junctions in the 3´ UTR.”*

*Regarding the question whether uORFs are also enriched in NMD-sensitive transcripts that have an intron in the 3´UTR: Of totally 1522 transcripts that are NMD-sensitive and have their stop codon not in the last exon, 445 transcripts had a uORF, which is 29%. Thus, as would have been expected, in these transcripts the EJC in the 3´UTR seems to be the dominating NMD-inducing feature and uORFs are not enriched in this category of transcripts compared to transcripts that are insensitive to NMD.*

2-	P7. "NMD targets specific RNA isoforms". With the overall approach to identify NMD targets they are probably including some indirect NMD targets as well. Those might be the NMD exon skipping events where the expression changes only in the gene (Fig 2E, black dots) but not in the transcripts. If that is the case, then it may not be surprising to see exon skipping as the main NMD-inducing type of event, because it's also the most common splicing event in normal genes. Can they comment on this possibility that NMD indirect targets confound analysis to inflate the relative frequency of exon-skipping as the NMD inducing event?

*Response: With this Figure, we intended to show that there is a general stabilization of transcripts upon NMD inactivation, as expected, and furthermore, that there are many cases where changes are detected at isoform but not at the gene level (green dots). This is a crucial message of our manuscript. In the analysis of NMD-inducing features (shown in fig. 4), we only included cases where the expression changes were observed at the transcript level.*

*With the experimental approach of inactivating NMD by knocking down NMD factors, indirect effects cannot be excluded. However, we attempted to minimize indirect effects (i) by minimizing the time of the knockdown and (ii) by employing rescue conditions. We have stated on p. 20 of the discussion the caveat that our approach inevitably will also include some indirect NMD targets. However, that the vast majority of the observed changes in alternative exons appear to be direct NMD targets is documented by the fact that most of the detected events (dots) lay in the upper right quadrant of the plot (i.e. the vast majority of alternative exons increased upon NMD inactivation and decreased upon NMD rescue). Indirectly regulated targets would be expected to exhibit an equal frequency between up-and down-regulated events, which is clearly not the case in our data.*

3-	P13 L13 - "we decided to address the longstanding question of whether NMD also targets endogenous mRNAs with long 3 UTRs, independently of exon junctions." [...]

Their data, and other previous analysis (e.g. PMID: 27618451) suggest that 3'UTR length seems not to be an NMD-inducing feature in human. However there are also prior studies with experimental work showing that the longer the 3' UTR, the higher degradation by NMD, even without 3' UTR EJC (although this is not as efficient as the isoforms having 3' UTR EJC), possibly due to the quantity of UPF1 protein that is bound to the 3' UTR. Can they comment on what would be the reason for having observed the effects of 3' UTR lengths previously? Perhaps the UTR-length triggering only happens in certain transcripts, but it is not a general rule. Is it possible that in Fig 4C, we only observe a significant difference in the first <2000 nt or so?

*Response: The effect of 3’ UTR length does not seem to be general, as our figure shows. It is possible that for some transcripts with specific lengths and perhaps with other properties the 3’ UTR length matters for NMD. A possible reason for finding NMD targets to have on average longer 3´UTRs than NMD insensitive mRNAs could be that in the past, the 3'UTR length has not been systematically addressed independently of the presence of EJCs in the 3´UTR. Moreover, short reads cannot unambiguously be mapped to specific isoforms with different splice sites in the 3'UTR, which could have been a confounding factor in these studies.*

*We do not think that we should repeat the analysis by selecting shorter length bins, because if there would be a correlation between the length of the 3'UTR and the sensitivity to NMD, the effect would be apparent in longer 3'UTRs.*

4-	Supp Fig 1A. what happened with SMG6 an dKD conditions? They obtained 75% less reads than in the other samples. Do they have replicates? I understood they have 3 replicates for the Illumina sequencing, but no replicates for the Nanopore. In these two conditions they detect a similar number of genes/transcripts as in the other samples/conditions (Fig.1C), so it might mean that the coverage is 75% lower if I understand correctly. Is this decreasing the reliability of the SMG6&_dKD results?

*Response: Indeed, in this experiment we obtained fewer reads. However, we did not see a difference in gene capture rate and, most importantly, the expression level quantifications were all done based on short-read data. Does neither the quality nor the depth of our analyses was not influenced by this one experiment yielding fewer reads than the others.*

5-	P19 L31: "it should be taken into consideration that many computationally predicted uORFs are in fact not translated and therefore do not stimulate NMD (52)." Could they consult a database with evidence of translation of various transcripts in human, such that they could filter out all the uORFS for which translation is not reported. This would help gain confidence that the higher differences in uORFs abundance in NMD-sens vs NMD-insens transcripts is due to translation of uORFs.

*Response: We thank the reviewer for this suggestion. As proposed, we consulted Ribo-Seq data of experiments performed in HeLa cells (Bohlen et al., Nat. Commun. 2020), intersected it with our computationally predicted uORFs and repeated our analysis by only considering uORFs for which there was Ribo-seq evidence indicating their translation. For approximately*

*1/3 of the predicted uORFs, the Ribo-seq data did not provide evidence for translation but since this applied equally to all transcript categories, our initial result remained essentially the same, except that the numbers and percentages are now lower by one third, and the conclusion remains unchanged.*

*We decided to include this new, refined analysis in the manuscript and have replaced Fig. 4D with the new results. This replacement of Fig. 4D required the following adjustments in the text (p.13): “...we identified computationally ORFs starting with an AUG and encoding at least five amino acids (15 nt) within the 5΄UTR and upstream of the main APPRIS-designated ORFs and included those in our analysis, for which a recently published ribosome profiling data set provided experimental evidence for their translation (57)” and in the legend of Fig. 4D: "Relative percentage of experimentally validated uORFs...". We also updated the material and methods accordingly (p.23): "To focus on uORF that are indeed translated, we intersected the computationally predicted uORFs with recently published RiboSeq data (57) and retained only those with experimental evidence for translation. This analysis was performed with zgtf of the ZARP pipeline (104)."*

6- could they comment on and/or quantitate whether the long-read sequencing technology is overall more important for the discovery of new NMD target isoforms (with very low gene expression and so missed thus far), or for the refinement of already known NMD targets?

*Response: The key in our study was to combine short-read and long-read data. The main advantage of long-read sequencing is in expanding the known transcriptome, allowing us to identify novel NMD targets (we identified 13% novel alternative exons in our study; Fig. 2C). As an example where only long-read sequencing allowed the identification of the NMD-sensitive transcript isoforms, we showcased UTP11 in Fig. 3B. Long-read sequencing identified for this gene two different previously unknown NMD-specific transcript isoforms, one arising by the inclusion of an extra exon in the 5´half of the transcript and one by the inclusion of an extra exon in the 3´ half. Based on short-read data only, it would not have been possible to distinguish if these alternative exon inclusion events occur on the same transcript or on distinct ones.*

*However, it must also be emphasized that long-read sequencing does not currently permit accurate quantifications because of limited sequencing depth, so we therefore think that the combination of the two can push the envelope of knowledge of NMD targets.*

Other remarks:

- regarding P8 L29 and P7 L51, clarification may be needed. From how I understood the text, the newly detected isoforms are in 686 of 8056 genes, which accounts for ~8% of the genes (or exon skipping events?) with no significant gene expression change. If that is not the case, which is the total number (and % total genes/transcripts) of newly identified isoforms? The text also mentions a 13%, but this seems to refer to the newly-identified splicing events.

*Response: The reviewer refers to the number of isoforms in genes for which no significant change in the expression levels was detected. To this number, the number of genes with differential expression after the depletion of SMG6 and SMG7 should be added (1488). The total number of genes with changes in the ratio of specific isoforms is therefore 2174. However, we cannot deduce any information for the total number of new isoforms but we only define the fraction of genes that show a different ratio of the isoform levels between the tested conditions. To the best of our knowledge, there is no published list of known NMD-sensitive isoforms based on experimental data to compare our results to.*

*However, prompted by the reviewer’s comment, we now deposited our transcriptome annotation in GEO alongside with the sequencing data and added a supplementary table (Table S1) with a list of the NMD-sensitive and insensitive transcripts and the corresponding genes for the analysis of the NMD-inducing features shown in Fig. 4.*

- P22 L21 - "the tool selects one start codon for each gene from the APPRIS (103) database of high-confidence transcript isoforms and then looks for a downstream in-frame stop codon in the transcripts annotated for the given gene" I presume all of the stops are normal stop codons (not PTC); if otherwise, consider rephrasing.

*Response: In this analysis, we used the start codon of the principal isoform as defined by APPRIS and we then deduced the CDS that derives from NMD-sensitive and insensitive isoforms and therefore the termination codon. All stop codons of the principal isoforms are the ones annotated from APPRIS and the ones of NMD-isoforms are annotated by our analysis.*

- References 85 and 93 appear duplicated.

*Response: We thank the reviewer for spotting this mistake and corrected it.*

- Page 4, line 25-26. Consider rephrasing sentence.

*Response: He have rephrased this sentence as follows:*

*"We also revealed that NMD targets canonically and non-canonically spliced mRNAs, indicating that NMD serves as a regulatory mechanism but also as a mechanism to rid the transcriptome of aberrantly spliced transcripts."*

- Fig. 1B. legend could be improved, e.g. they mention the blue and green lines, but not the purple lines. In the lower part (representation of the short-reads expression level patterns of NMD-sensitive exons) - I found it a bit confusing why if they are NMD-sensitive exons, in the right plot they are downregulated under NMD KD conditions. I suppose that the each

plot represents a different isoform of the same transcript: the isoform on the left contains a NMD-sensitive exon and the isoform on the right does not contain this exon. They might want to explain.

*Response: We have clarified the legend of Fig. 1B as follows:*

*(B) Upper part: Schematic representation of how long and short-read sequencing are combined to identify endogenous NMD-sensitive mRNA isoforms in human cells. Boxes denote exons (NMD-inducing exons in blue,), green lines denote long and short sequencing reads, long purple lines denote long reads that correspond to NMD-sensitive isoforms, short blue lines denote short reads that map to exons of NMD-sensitive isoforms. Lower part: Representation of the short-reads expression level patterns of NMD-sensitive exons. NMD-sensitive isoforms can occur by exon inclusion or exon exclusion and the patterns of changes of the expression levels to opposite directions are taken into consideration.*

- They could explain better what the NMD KD rescue condition entails. They show it in figures and refer to it through the text, but give no explanation how they do the rescue in the methods. We just know from the text that they overexpress the NMD factors; more experimental detail would be appreciated.

*Response: The rescue experiments that are described in our manuscript were analyzed in our previous study (Colombo et al., 2017). To clarify this point we included the following sentence in Material and methods (p.21):*

*“The rescue experiments that were used to produce short read sequencing data are described in (38).”*

- Fig. 2E - in the legend they should state which two conditions they are comparing (I think it's dKD vs control).

*Response: Indeed, we used dKD versus control and we specified this now in the legend of Fig. 2E. The legend was complemented as follows:*

*“(E) Scatter plot depicting the log2 fold change (log2FC) of NMD-sensitive transcripts (y-axis) versus the log2FC between dKD and CTR of their corresponding genes (x-axis) showing alternative exon events (Ν= 8'056) based on SMG6/SMG7 double KD, CTR KD and SMG6 Rescue conditions.”*

- Fig. 4D - they claim that NMD-sensitive transcripts have more uORFS than NMD-insensitive transcripts (in the text and in the discussion). According to Fig. 4d, it is 35% vs 45%. I think they should mention these percentages in the text. From reading the text, one gets the impression of striking differences, but in reality the differences are subtle. (described by the word "evident", which is, admittedly, technically not wrong for a 10% difference).

*Response: We have re-phrased the corresponding sentence and add the requested numbers to enhance clarity. The sentence now reads (p.13): “While no difference in the fraction of*

*mRNAs with uORFs can be detected between all NMD-sensitive and insensitive mRNAs, an enrichment among NMD-sensitive mRNAs becomes evident when only mRNAs are considered that do not have exon-exon junctions in their 3´UTR (35.5% versus 24.9%; Fig. 4D).*

- Fig. 5 - besides the individual dots, it may be helpful to depict the distribution of the SRSF group (pink) to highlight the different behaviour from the rest of NMD splicing sites.

*Response: The SR proteins are already highlighted in this plot. It is not clear to us what additional changes are proposed by the reviewer.*

- Discussion is a bit verbose, and reads more like a summary of the paper. I would consider dropping some parts e.g. pg.18 line 27: they explain again the 50 nts rule (redundant).

*Response: As suggested, we deleted the mentioned part in the discussion. However, given that the article is intended for a broader audience, including the Long-read sequencing community, that may not be familiar with NMD, we think that the information given in the discussion is essential for better understanding the broader context and impact of our findings.*

**Second round of review**

**Reviewer 1**

The authors have performed a good revision and have addressed the minor concerns and suggestions that were raised during the first round of review. As such, it is my view that the revised manuscript is improved and that this paper is now acceptable for publication in Genome Biology.

**Reviewer 2**

The authors have satisfactorily addressed my comments (which were minor to begin with). I can recommend publication.
